# Basal ganglia as an fMRI motor neurofeedback target in Parkinson’s disease

**DOI:** 10.1007/s10484-025-09747-5

**Published:** 2025-11-19

**Authors:** Halim I. Baqapuri, Anneke Terneusen, Michael Luehrs, Judith Peters, Mark Kuijf, Rainer Goebel, David Linden, Neuripides Study Group, Andres M. Lozano, Josef Mana, Bechir Jarraya, Ricardo Loução, Martin Kocher, Veerle Visser-Vandewalle, Tolga Cukur

**Affiliations:** 1 Faculty of Health, Medicine and Life Sciences, Mental Health and Neuroscience Research Institute, Maastricht University, Universiteitssingel 40, 6229 ER Maastricht, The Netherlands; 2 Department of Cognitive Neuroscience, Faculty of Psychology and Neuroscience, Maastricht University, Universiteitssingel 40, 6229 ER Maastricht, The Netherlands; 3 Brain Innovation, Oxfordlaan 55, 6229 EV Maastricht, The Netherlands; 4 Department of Neurology, Maastricht University Medical Center, Maastricht, The Netherlands; 5 Division of Neurosurgery, Department of Surgery, University of Toronto, Toronto, ON Canada; 6 Department of Neurology and Centre of Clinical Neuroscience, First Faculty of Medicine and General University Hospital in Prague, Charles University, Prague, Czech Republic; 7 Cognitive Neuroimaging Unit, CEA, INSERM, NeuroSpin Center, Université Paris-Saclay, 91191 Gif-sur-Yvette, France; 8 Department of Stereotactic and Functional Neurosurgery, Faculty of Medicine, Centre for Neurosurgery, University and University Hospital of Cologne, Cologne, Germany; 9 Department of Electrical-Electronics Engineering, National Magnetic Resonance Research Center (UMRAM), Bilkent University, Ankara, Turkey

**Keywords:** Parkinson’s disease, Basal ganglia, Neurofeedback, Real-time fMRI, Deep brain stimulation, Motor imagery

## Abstract

Parkinson’s disease (PD) is a progressive neurodegenerative disorder characterized by motor impairments. While pharmacological treatments offer symptom alleviation, their long-term effectiveness is insufficient. Deep Brain Stimulation (DBS) is a neurosurgical treatment that targets brain pathways to alleviate motor symptoms in PD. It is a highly invasive procedure and carries associated risks. This prompts investigation of non-invasive alternatives, such as real-time functional Magnetic Resonance Imaging (rt-fMRI) neurofeedback (NF). This work investigates the feasibility of using the basal ganglia, more specifically the putamen, a key structure in the motor network, as a potential NF target region. Two rt-fMRI studies were conducted: (i) Twelve healthy individuals participated in a single-blind, crossover study involving one MRI session targeting the putamen and the supplementary motor area (SMA) in separate runs. (ii) Twelve PD patients followed the same protocol but with three MRI sessions. We investigated whether participants could learn to voluntarily control brain activity through NF training. The PD patients successfully recruited the putamen during NF-reinforced motor imagery, which was also found at trend level in the healthy participants. We found no learning effect and no difference in putamen activation when it was directly targeted versus when the target signals came from the SMA. Overall, widespread cortical and subcortical areas involved in motor control were activated during neurofeedback. This study demonstrates for the first time that PD patients can modulate putamen activity through NF training, supporting its potential as a non-invasive neuromodulation target. This opens opportunities for integrating invasive and non-invasive neuromodulation for PD treatment.

**Supplementary Information:**

The online version contains supplementary material available at 10.1007/s10484-025-09747-5.

## Introduction

Parkinson’s disease (PD) is a progressive neurodegenerative disorder primarily characterized by motor impairments and affecting both motor and non-motor functions. It is one of the most common and fastest growing neurodegenerative disorders, having a prevalence of 1–2 per 1000 and affecting 1% of the population above 60 years of age (Lamptey et al., 2022; Tysnes & Storstein, 2017). According to the PD fact sheet of the world health organization, the prevalence of PD has doubled in the past 25 years, estimating at 8.5 million individuals globally diagnosed with PD^1^. While dopaminergic pharmacological treatments have proven effective in alleviating some of the symptoms associated with PD (Connolly & Lang, 2014), they are not without limitations such as response fluctuations and dyskinesias associated with increased disease duration (Beaulieu-Boire & Lang, 2015; Hametner et al., 2010). Thus, other approaches that target the underlying neuropathology of PD are of great interest in translational research.

Most research on the pathophysiology and treatment mechanisms of PD focuses on the basal ganglia (Obeso et al., 2008). In this context, deep brain stimulation (DBS) has emerged as a widely utilized surgical intervention for targeting neural pathways in PD (Bucur & Papagno, 2023; Mahlknecht et al., 2022). For PD, DBS involves the implantation of electrodes in specific brain regions, most commonly the subthalamic nucleus (STN) or globus pallidus internus (GPi). These electrodes are then connected to a stimulator which can send electrical pulses in specific sequences, and in some cases the resulting activity in the brain region can also be read back by these electrode probes (Little et al., 2013). This approach is most effective in ameliorating motor symptoms. However, DBS is an invasive procedure, necessitating brain surgery and continuous hardware maintenance. It also carries inherent risks and can elicit side effects, including cognitive and behavioral changes (Fenoy & Simpson, 2014; Moldovan et al., 2015; Saleh & Fontaine, 2015). As such, there is growing interest in exploring non-invasive methods that can replace or complement DBS for managing PD symptoms.

Advancements in functional magnetic resonance imaging (fMRI) have allowed researchers to study neural processes in humans at high spatial resolutions, non-invasively. Real-time fMRI (rt-fMRI) enables the measurement of whole brain functional activity online, and can be utilized to provide feedback to the participants for self-regulation training (Sitaram et al., 2017). This methodology is called neurofeedback (NF). NF enables patients to develop personal strategies that are most effective in regulating activation patterns in specific brain areas or networks. Participants can learn to voluntarily control these activation patterns, by receiving real-time information about their functional brain activity from a specified region of interest (ROI). Therefore, it can provide an individually tailored intervention for neuromodulation (MacDuffie et al., 2018; Sorger et al., 2018; Stoeckel et al., 2014). One key aspect in the design of rt-fMRI NF studies is the choice of an appropriate ROI. Since ROIs are the targeted region for regulation using NF training, they are often chosen based on their involvement in the cognitive process under study. For example, Linden and colleagues showed improvement in symptom scores of depression patients after NF training, using target regions associated with positive emotions such as ventrolateral prefrontal cortex and the insula (Linden et al., 2012). Generally, in patient studies a target region is selected that is associated with dysfunction in the studied disorder (Mehler et al., 2019; Paret et al., 2016; Zweerings et al., 2019). With regard to motor activations and related behavioral measures, one of the most studied ROIs in NF is the supplementary motor area (SMA) (Al-Wasity et al., 2021; Hampson et al., 2011; Scharnowski et al., 2015). It is often chosen because of its established role in the motor network and its involvement in planning and initiation of motor activities (Nachev et al., 2008).

As rt-fMRI NF shows promise of individualized training via self-regulation of affected brain regions, it has also been explored as a training method in PD. One of the first works to demonstrate the application of fMRI NF in PD was by Subramanian and colleagues. They showed feasibility of the NF method by showing active recruitment of the SMA during the training as well as behavioral outcome improvements, such as the motor scale improvement of the Unified Parkinson’s Disease Rating Scale (Subramanian et al., 2011). Following this, Buyukturkoglu and colleagues also demonstrated recruitment of the SMA, but with no clinical outcome improvement, however, in their work only one PD patient was included (Buyukturkoglu et al., 2013). More recent research has replicated the feasibility of fMRI NF in PD, however, there are only a handful of studies demonstrating this (for a review see: (Anil et al., 2021)). Importantly, no previous study has targeted the basal ganglia directly in PD patients, although several structures within this region have been used as primary targets in studies using DBS, which is one of the most effective neuromodulation treatments for PD. To bridge this gap in NF research, we aimed here to establish the basal ganglia as a viable target region for fMRI-based NF training.

Basal ganglia and more specifically the STN and the GPi were our initial targets as potential ROIs for NF due to their documented dysregulation in PD (Kühn et al., 2009; Vila et al., 1997), as well as being common DBS implantation targets. We conducted a preliminary study to evaluate the technical setup for detecting neural activity during a NF paradigm. The findings from this study indicated that participants experienced relative difficulty in recruiting the two target regions during NF. In contrast, neural activity in the putamen showed greater responsiveness, and more effectively captured the NF training within the context of a motor-based paradigm. This outcome is not surprising since the putamen is a key structure within the basal ganglia responsible for regulating motor activities, whose function can be disrupted in PD (Fearnley & Lees, 1991; Kish et al., 1988). The putamen is also directly linked with the substantia nigra, a key brain region responsible for the production of dopamine, a neurotransmitter that plays a crucial role in regulating various systems within the central nervous system, particularly those involved in motor control (Sonne et al., 2024). The substantia nigra pars compacta projects to the dorsal and posterior parts of the putamen. In fact, the degeneration of the putaminal dopamine is considered to directly affect motor effort in PD patients (Banwinkler et al., 2024). Consequently, the putamen has been shown to be functionally underactivated in PD (Herz et al., 2021).

These findings and considerations led to the putamen being selected as a candidate of interest for NF interventions in PD. Moreover, given its proximity to the DBS targets and its larger volume compared to most other basal ganglia regions, the putamen makes for a practical NF target within the subcortical motor control circuit. We designed two studies to investigate the feasibility of the putamen as a NF ROI and compare its recruitment during training to the SMA, which is an established cortical motor NF target ROI. Our goal in the first study was to establish basic feasibility of the methodology in recruiting subcortical regions for NF. We intended to identify any technical issues that would arise, minimize any risk associated with the method and optimize the protocol prior to applying it in a patient cohort. Therefore, in the first study, we focused on implementing our methodology in a healthy cohort during a single MRI training session. In the second study we transferred our method to the patient cohort in a three-session experimental design. This enabled us to investigate any potential patient specific responses and was intended to test the feasibility of the putamen for future interventional trials. We hypothesized:

1. The participants would be able to activate the putamen during NF training using motor imagery.
2. The participants would learn to effectively self-regulate the putamen activity using the feedback provided.
3. We would observe similar activation patterns in both SMA and putamen, although, they would differ in their activation amplitudes. The rationale for this expectation was that both ROIs are part of the motor control network.

## Methods

### Participants

#### Healthy cohort study

We recruited twelve healthy volunteers (mean age: 27.3 ± 4.2 years; 9 females) to the study. The participants were recruited using Maastricht University’s internal online research participation system and personal contacts.

All participants reported no acute psychiatric, neurological or medical disorders, nor contraindications to MRI. Vision was normal or corrected to normal. The experiment was designed according to the Code of Ethics of the World Medical Association (Declaration of Helsinki, 2008). The study protocol was approved by the Ethics Committee of the Faculty of Psychology and Neuroscience of Maastricht University. After complete description of the study to the subjects, written informed consent was obtained.

#### Patient cohort study

We recruited twelve volunteers (mean age: 60.5 ± 8.0 years; 6 females) with a PD diagnosis according to the Movement Disorders Society diagnostic criteria (Postuma et al., 2015) to the patient cohort study. Participants were recruited through the Parkinson Vereniging, which is a Dutch Parkinson’s association, and through the Maastricht University Medical Center. All participants were assessed to be on the Hoehn and Yahr scale stage 1 – 2. All participants continued to take their regular medication following their schedule during the study.

All participants reported no acute psychiatric, neurological (apart from PD) or medical disorders, nor contraindications to MRI. Vision was normal or corrected to normal. The experiment was designed according to the Code of Ethics of the World Medical Association (Declaration of Helsinki, 2008). The study protocol was approved by the Ethics Committee of Maastricht University Medical Center. The study was pre-registered at clinicaltrials.gov (NCT05627895). After complete description of the study to the subjects, written informed consent was obtained. One patient was later (after the experiment) reclassified as having an atypical dopamine deficiency.

### Experimental design

#### Healthy cohort study

**Fig. 1:**
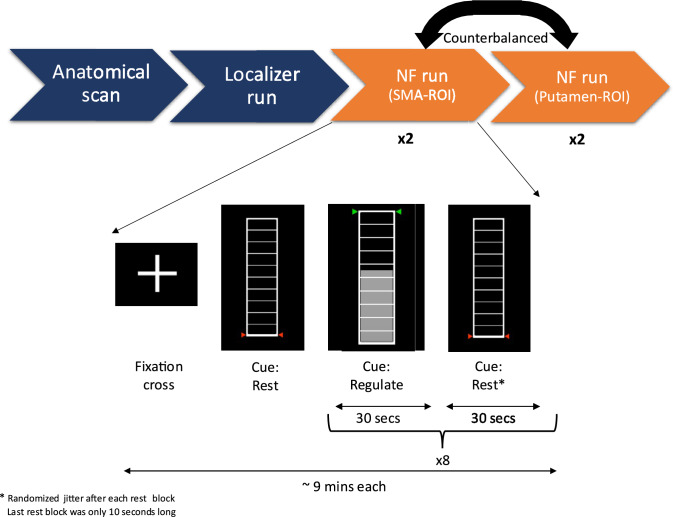
The design of the NF session, NF runs and block trials during the MRI measurement. The total measurement time in the scanner is approximately 60 min. The green/red arrows function as a cue and an activation target for participants to achieve during the full run

We conducted an experimental study assessing the ability to self-regulate components of the brain’s motor control network by NF in a single MRI session. After screening, participants were invited to the MRI session, which was about two hours long. This included approximately one hour of scanning time with the remaining time used for preparation and debriefing. At the start of the MRI session, all participants received standardized instructions to upregulate brain activity using motor imagery with NF. Each participant was free to choose their cognitive strategies, the only guiding instruction they were given was “imagine performing actions with movement”, as the target ROIs were part of the motor network. Furthermore, they were instructed to refrain from executing movements and it was explained to them that the feedback being displayed reflects their mental activity with a few seconds of delay due to the hemodynamic response. when viewing their feedback on the display. The MRI session consisted of six measurements (Fig. 1): one anatomical run, one localizer run, and four NF runs. The NF runs were divided into two conditions: SMA-ROI and putamen-ROI. In the SMA-ROI condition the participants received feedback from the SMA and during the putamen-ROI condition the feedback was given from the putamen. Two of the four NF runs were in the SMA-ROI condition and two were in the putamen-ROI condition. Runs in each condition always took place consecutively, i.e., either the first two runs were SMA-ROI and the last two runs were putamen-ROI or vice versa. This was counterbalanced among the participants in a pseudorandomized manner. The participants were blind to which area was targeted in each run.

The anatomical run was used to standardize the individual brain space into the Montreal neurological Institute (MNI) space and for defining the ROIs at a later stage. The localizer run was used to identify participant-specific ROIs for the NF runs. This run consisted of eleven resting blocks and ten activation blocks total, where each activation block was 30 s long. In five of the activation blocks participants were asked to physically open and close their hands, whereas in the remaining five they used motor imagery. During the entire localizer run, the participants saw an empty thermometer and did not receive any feedback. After the localizer run, an activation map was generated by contrasting all ten activation blocks against the rest blocks. The AAL atlas anatomical definitions of the SMA and the putamen were used to circumscribe the regions on this activation map, from which the individualized ROIs were subsequently created.

The Turbo-BrainVoyager (TBV) software (version 4.2.0; BrainInnovation B.V., Maastricht, The Netherlands) plugin “Best Voxel Selection” was used to identify the best activated 300 voxels in each circumscribed region. This generated the two ROIs for the NF runs. This method allowed us to define an individualized ROI within the putamen and SMA for each participant, based on their unique activity patterns during the localizer run. Apart from the targeted ROI, all NF runs were identical. They consisted of seventeen blocks: eight regulation blocks and nine resting blocks, where all regulation blocks were 30 s each. The participants were instructed to upregulate (increase) their brain activity during the regulation blocks and achieve the maximum level of the thermometer (Fig. 1). Once they had achieved this, the participants were instructed to keep it at this level as long as possible. For the resting blocks, the participants were instructed to not think about anything specific and to let their mind wander freely. After each NF run, the participants were asked what cognitive strategies they had used during the regulation blocks.

During all functional runs, the participants viewed a thermometer bar with ten divisions. The thermometer was white and displayed on a black background. The fill of the thermometer was grey colored, and the level of the fill represented activation strength in the ROI, with the maximum height of the thermometer scaled to 1% blood-oxygen-level-dependent (BOLD) signal change. The thermometer level was updated each repetition time (TR) of the functional run (i.e., 1 s). The display of the thermometer level was updated smoothly, i.e., the BOLD signal change of the previous TR and the current TR were used to interpolate between the two values and display a smooth transition of the thermometer fill. Throughout the functional runs, the thermometer either had green or red symbols on its sides, indicating if it was a regulation or a resting block. Additionally, at the start of each block, participants saw a written instruction and a colored symbol, displayed for one second, informing them about the start of a new block.

#### Physiological controls

To enhance the robustness of our experimental design and minimize potential confounding variables, we introduced two additional controls for physical movement into the paradigm. In our cohort of twelve participants, six were controlled for physical body movement using electromyography (EMG) measured on their dominant hand, and six participants were assessed using an eye tracking system for controlling oculomotor differences between the activate and rest conditions of the NF runs. For the EMG measurements, a three-electrode arrangement was used. Two electrodes were placed on the dorsal side of the dominant hand of the participant, following a setup commonly used in motor-evoked potential studies. The positive electrode was proximal to the index digit, placed behind the metacarpophalangeal joint, and the negative electrode was placed proximal to the wrist. The ground electrode was placed on the lower leg of the participant, proximally above the talocrural joint. With this arrangement we captured activity in the dorsal interossei, particularly the first dorsal interosseous muscle. The eye-tracking system was affixed onto the MRI scanner head coil and measured eye movement from the center of sight. The center was calibrated at the start of each NF run using a fixation cross at the center of the display. For the six participants with eye tracking, the resting blocks were modified so that they could also view the ROI activity on the thermometer bar.

#### Patient cohort study

**Fig. 2:**
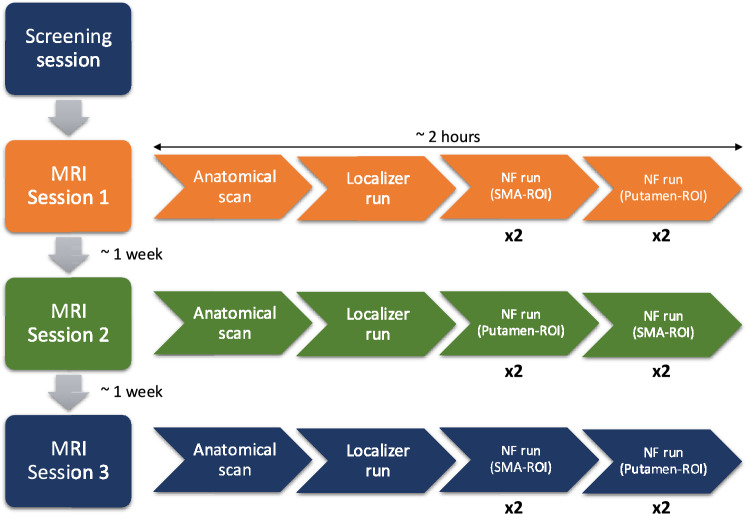
The patient cohort study invited participants to three sessions of NF training approximately one week apart. Participants were blind to the condition and the order of the runs reversed each week

The experimental design for this study followed the healthy cohort study with two major differences:

1. The number of total MRI sessions was increased to a maximum of three. Participants were invited for three NF training sessions, which were approximately 1 week apart (Fig. 2). In certain cases, due to scheduling conflicts, this delay between measurements could not be followed. If during the first MRI session, the participant was assigned the SMA-ROI condition followed by the putamen-ROI condition, in the following week this was reversed and vice versa. This was counterbalanced among the participants in a pseudorandomized manner. The participants were blind to the target areas.
2. Only the EMG was recorded for all twelve participants.

### Materials and data acquisition

A Panasonic PT-EZ570U projector (Panasonic, Secaucus, NJ, USA) was used to display the paradigm to the participants. The image was projected onto an acrylic (DA-plex DA-100 WA) screen positioned towards the head opening of the scanner bore. The screen was 47 cm diagonally, displayed the feedback on a native 1900*1200 pixel resolution, and was viewed by the participant using a mirror. The participants’ eyes were approximately 5–10 cm from the mirror, and the distance between the mirror and the screen was approximately 60 cm.

An Avotec Realeyes (RE5721) Binocular Eyetracking system (Avotec Inc., Stuart, FL, USA) was used for the sessions with eye tracking and placed on top of the scanner coil. This system was connected to a dedicated computer where the ViewPoint EyeTracker® (version 2.9.5.311, Arrington Research, Scottsdale, AZ, USA) software recorded the eye movement data. For the EMG measurements a BrainAmp ExG system (Brain Products GmbH, Gilching, Germany) was used and the data was recorded using the BrainVision Recorder software (version 1.23.0003, Brain Products GmbH, Gilching, Germany) on a dedicated computer.

A 3-T Siemens-Prisma fit scanner with a 64-channel head coil was used for the measurements. Anatomical images were acquired with a magnetization prepared rapid acquisition gradient echo sequence (TR = 2250 ms; TE = 2.21 ms; FoV = 256 mm; 1 mm isotropic voxel size; GRAPPA factor = 2). Each functional run was acquired using a multiband echo planar imaging sequence with the following parameters: TR = 1000 ms; echo time = 31 ms; matrix = 90 × 90; slices = 48 with multiband factor = 4; flip angle = 62°; and transversal slice orientation. The localizer run had 636 volumes whereas the NF runs had 512 volumes.

### Measures and statistical analysis

For updating the display of the thermometer bar, reconstructed DICOM images were transmitted in real-time from the scanner computer to a dedicated computer with the TBV software. TBV was used for real-time online pre-processing and analysis of the BOLD signals. Rigid body transformations were used for motion correction relative to the first functional volume acquired from the localizer run. A 4-mm full width at half maximum (FWHM) kernel was applied for smoothing of functional data. For feedback presentation during the NF runs, the average percentage signal change (PSC) value was extracted from the respective ROI in TBV and transmitted to a separate computer dedicated for displaying the feedback in real-time. This was done via a TCP/IP network protocol over the local network to minimize any delays in transmission. The PSC value was then normalized to the display thermometer such that 1% PSC would fill the bar completely.

For the offline analysis of MRI data, we used BrainVoyager (BV, version 22.2, Brain Innovation, Maastricht, the Netherlands). To investigate the specific effects of regulation in the putamen and the SMA, ROI analyses were carried out. The AAL atlas was used to anatomically define the putamen and the SMA. During the localizer run, the most activated 300 voxels (bilaterally) were selected within these two anatomically defined ROIs. These voxels formed the individual ROIs for the participants. Intensity inhomogeneity correction for anatomical images was applied before transforming them into MNI space. Standard pre-processing steps including scan-slice time correction with cubic spline interpolation and intra-session motion correction relative to the first volume of the first functional run with trilinear detection and sinc interpolation were applied. Realigned functional images were subsequently registered to the corresponding anatomical image, and spatially smoothed with a 7 mm Gaussian full width at half maximum kernel. To account for T1-saturation effects, the first 10 volumes of each scanning session were not considered. The acquired images were subjected to a general linear model (GLM) for each subject. On the group level, ROI GLM analyses were performed for both ROIs (p_FWE_ < 0.05). T-tests were performed to assess the specific effects of regulation in both ROIs.

We used BrainVision Analyzer (version 2.2, Brain Products GmbH, Gilching, Germany) to preprocess and analyze the EMG data. MR-Artifact correction was applied to all runs using default values; data was downsampled from a sampling frequency of 5000–250 Hz, a finite impulse response filter was used with a cut-off frequency of 40 Hz and the data was synced to the scanner using an MR trigger. The data was then root mean squared and segmented into 1 s intervals using the MR triggers. It was then exported to MATLAB (Release R2022b, version 9.13; MathWorks, Inc., Natick, MA, United States) for plotting and analysis. Paired sample t-tests with a threshold of *p* < *0.05* were computed between the localizer hand movement blocks and (i) localizer imagery blocks, (ii) NF blocks and (iii) rest blocks.

The ViewPoint EyeTracker® recorded data in a text file, which was parsed into MATLAB and processed. The data was first synchronized to the MRI scan using volume triggers recorded in the text file. The data was sampled at 60 Hz. We extracted the corrected gaze variable which includes standard eye tracking preprocessing (i.e., Exponential moving average smoothing with 4 timepoints) and pupil segmentation already performed. Since the thermometer only moved in the vertical direction, the data from the vertical axis of the eyes was extracted. We then defined a threshold for the vertical eye movement. If the gaze wandered more than 30% of the distance from the center of the screen in either vertical direction, we considered that as significant gaze movement. The total number of times that each participant had significant gaze movement was then recorded for each block (NF and rest) and averaged across participants. A paired sample t-test with a threshold of *p* < *0.05* was performed between the NF and rest blocks.

To evaluate the learning effect on NF performance, a full factorial repeated-measures analysis of variance (ANOVA) was performed. The 2 factors used were the effect of session and the effect of ROI. Lastly, we also performed an exploratory whole brain analysis to investigate the spatial activation patterns during the NF training. T-maps for the contrasts of interest at the group level were created with a voxel-wise thresholding of *p* < *0.05*.

## Results

### Healthy cohort study

#### ROI performance

We performed an ROI analysis for each of the target regions (putamen and SMA) in the runs in which they were the target ROIs. We compared the activation during NF blocks with that during resting blocks of the same runs (NF > Rest). The putamen showed trend-level activation during the NF blocks (*T*(11) = 2.18, *p* = 0.052) when the putamen was targeted, whereas SMA activation was non-significant in the SMA-ROI runs (*T*(11) = 1.438, *p* = 0.18). Since both regions are part of the motor control network, we also looked at putamen and SMA activations when the region was not the target ROI, i.e., putamen activation during the SMA-ROI runs and vice versa. In this contrast, the putamen was not activated significantly when SMA was targeted (*T*(11) = 1.07, *p* = 0.31) whereas the SMA was significantly activated during the NF blocks targeting the putamen (*T*(11) = 2.309, *p* = 0.041). Finally, we wanted to compare the performance of the putamen during both conditions and defined a group contrast for putamen runs compared to the SMA runs. The putamen was not significantly more activated in the putamen-ROI runs than in the SMA-ROI runs (*T*(11) = 1.22, *p* = 0.25). These findings have been summarized in Table 1.

**Table 1:** Summary of the group level ROI analyses for the putamen and the SMA regions in the healthy cohort

ROI	Putamen Runs > Rest	SMA Runs > Rest
Putamen	T(11) = 2.18, *p* = 0.052	T(11) = 1.07, *p* = 0.31
SMA	T(11) = 2.309, *p* = 0.041	T(11) = 1.438, *p* = 0.18

	Putamen runs > SMA runs
Putamen	T(11) = 1.22, *p* = 0.25

#### Physiological controls

**Fig. 3:**
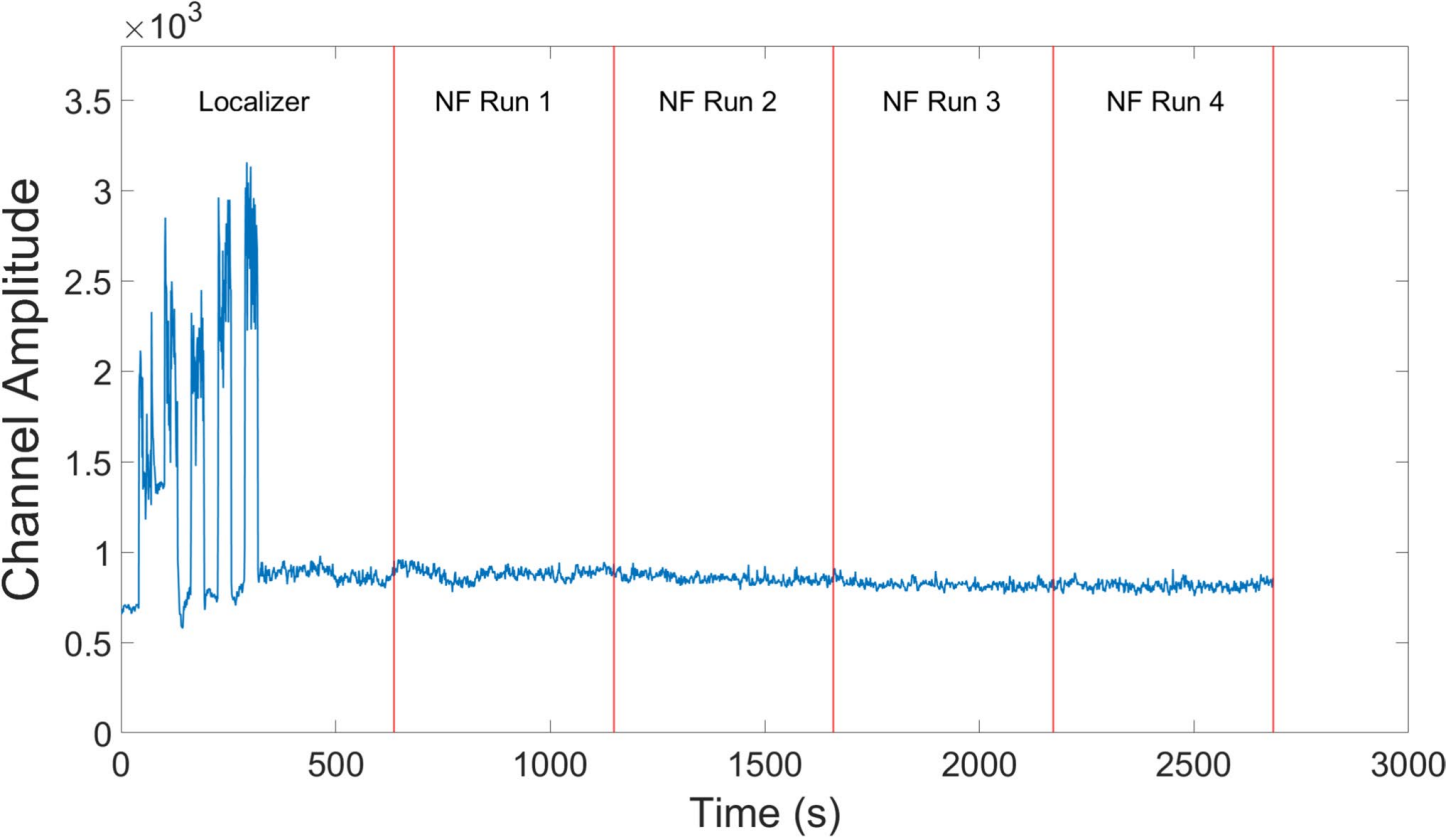
Averaged group EMG activations over the MRI session. The initial peaks correspond to the first five blocks of the localizer where the participants were asked to move their hands in a squeezing motion. The remaining five blocks of the localizer run as well as all the NF runs only used motor imagery as an activation strategy

**Fig. 4:**
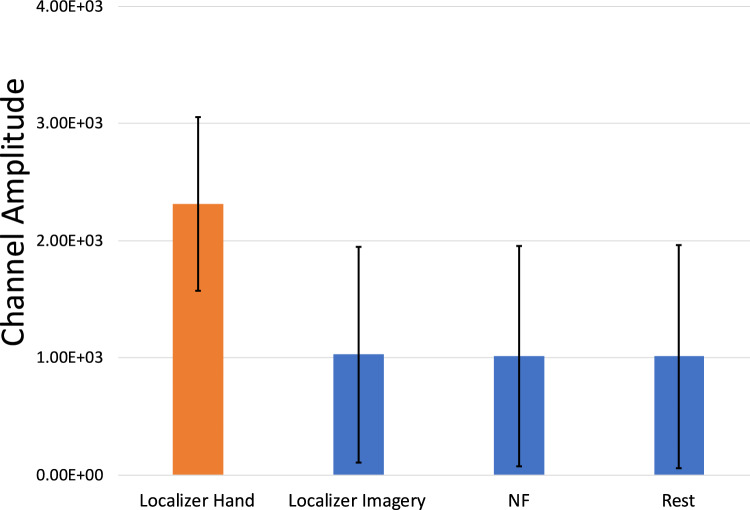
Average EMG activations over the different blocks within the localizer and NF runs for the healthy cohort. Error bars represent standard deviation

**Fig. 5:**
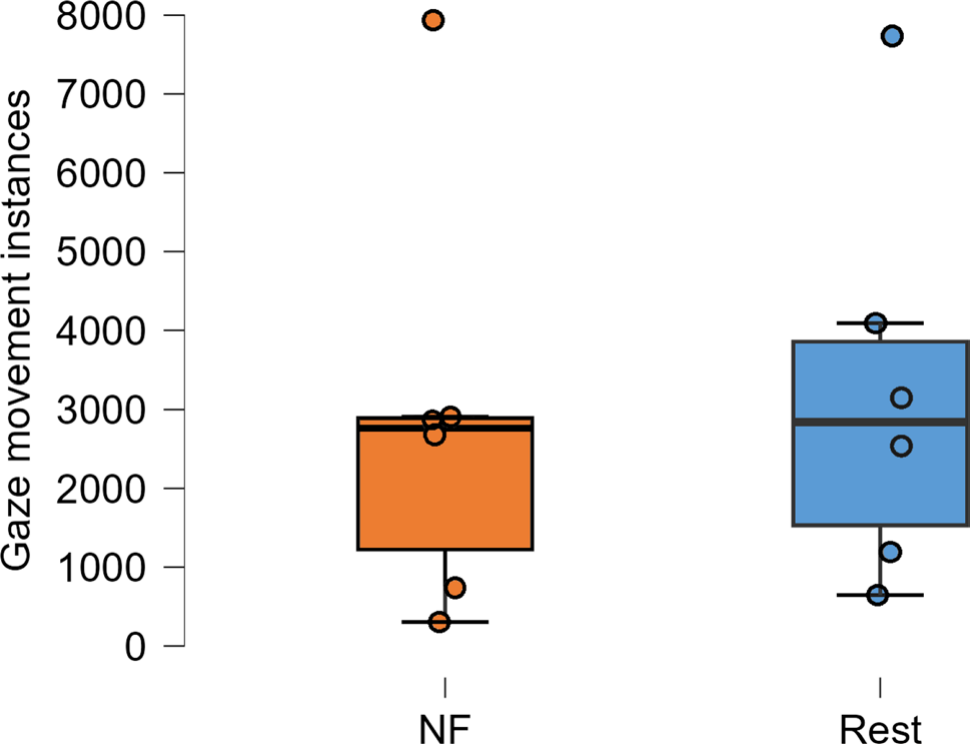
Average number of vertical significant gaze movement for the six participants with this movement control. The gaze movements were counted for both blocks in all runs and then averaged across participants

EMG activations were extracted per participant per session and averaged across participants. One participant had incomplete data due to a technical error during recording of the EMG, so the EMG activation plot for the MRI session is only averaged over 5 participants (Figs. 3,4). To determine if there was a difference in the movement between the localizer and NF tasks, we separated the EMG data into blocks in MATLAB and then compared their average amplitudes. Importantly, there was no difference between the NF blocks and the resting blocks (*t*(4) = 0.78, *p* = 0.48). However, there was trend-level increase of the EMG activity in the physical hand movement condition compared to the imagery condition in the localizer run (*t*(4) = 2.62, *p* = 0.059) and compared to the NF blocks (*t*(4) = 2.57, *p* = 0.062). When looking at the eye movement data for the remaining six participants (Fig. 5), we found that the vertical gaze movement was not significantly different between the NF and the resting blocks (*t*(5) = − 1.51, *p* = 0.19).

#### Event related averages

**Fig. 6:**
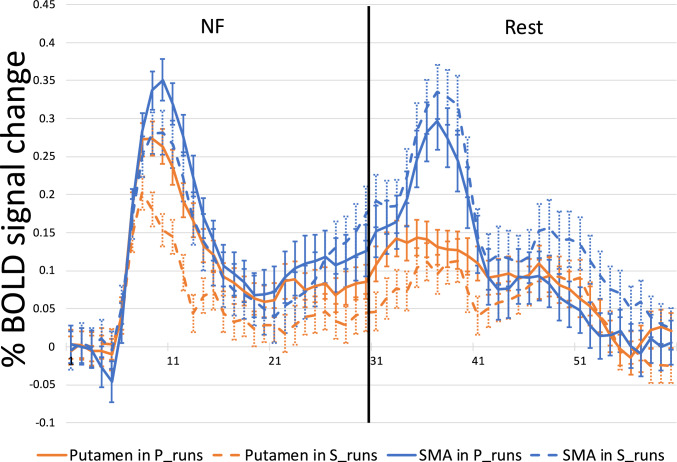
Percentage BOLD signal change during the NF blocks and the resting blocks for the healthy cohort. X axis represents seconds from onset of the neurofeedback block. The blue line indicates the putamen time course during the putamen-ROI runs. The yellow line indicates the putamen time course during the SMA-ROI runs. The orange line shows the SMA time course during the putamen-ROI runs while the green line shows the SMA time course during the SMA-ROI runs. The error bars represent standard error of the mean

To study the NF modulation across runs, we extracted the Event Related Averages (ERA) for the putamen and SMA regions in both conditions (Fig. 6). The NF and rest blocks both lasted 30 s. ERAs were extracted per participant per run and subsequently averaged across participants.

#### Cognitive strategies

After each NF run, the participants were asked to report the cognitive strategies they employed during the NF training. Their responses were recorded and broadly categorized into the following categories:

1. Exercise/Full body sport: This involved participants imagining performing physical exercises or engaging in sports that require full body exertion, such as running, swimming, playing football or working out at the gym.
2. Walking/Moving: In this strategy, the participants imagined general everyday movements such as walking, going to the store, and moving around the house.
3. Hand usage: This involved participants imagining or mentally rehearsing motor tasks or activities that primarily involve the hands, such as preparing food, gripping objects, or manipulating tools.
4. Observing movement: This strategy involved participants imagining themselves observing or watching others perform movements rather than engaging in the action themselves.
5. Speech: In this strategy, participants mentally simulated speaking or engaging in verbal communication.
6. Dancing: Participants employing this strategy mentally rehearsed or simulated dancing.

**Fig. 7:**
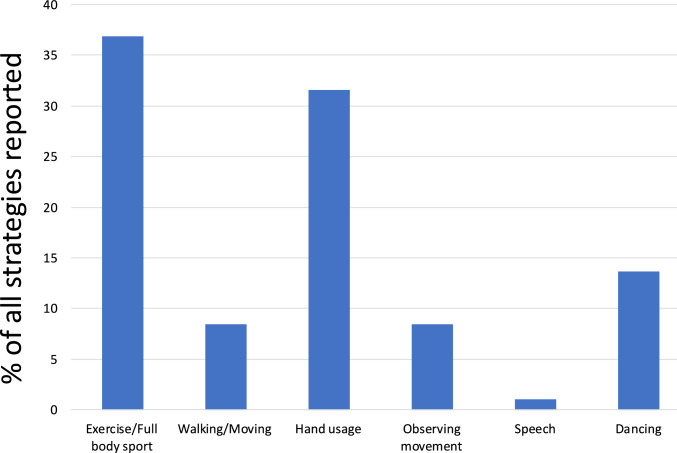
Distribution of self-reported cognitive strategies employed by healthy participants during the NF training runs

The distribution of these strategies is presented in Fig. 7, which visualizes each category as a percentage of all strategies reported.

### Patient cohort study

#### ROI performance

Similar to the analysis of the health participants, we performed an ROI analysis for each of the putamen and the SMA regions in their respective runs. We compared the activation during NF blocks with that during resting blocks of the same runs (NF > Rest) The putamen was significantly activated during the NF blocks (*T*(11) = 2.63, *p* = 0.023), whereas the SMA failed significance (*T*(11) = 1.83, *p* = 0.094). We also looked at putamen and SMA activations when the region was not the target ROI, i.e., putamen activation during the SMA-ROI runs and vice versa. In this contrast, the putamen and the SMA regions both showed trend-level activation [Putamen: *T*(11) = 2.07, *p* = 0.063; SMA: *T*(11) = 2.07, *p* = 0.063]. Finally, we wanted to compare the performance of the putamen during both conditions and defined a group contrast for putamen runs compared to the SMA runs. The putamen was equally activated in the putamen-ROI and SMA-ROI runs (*T*(11) = − 0.27, *p* = 0.80). These findings have been summarized in Table 2.

**Table 2:** Summary of the group level ROI analyses for the putamen and the SMA regions in the patient cohort

ROI	Putamen Runs > Rest	SMA Runs > Rest
Putamen	T(11) = 2.63, *p* = 0.023	T(11) = 2.07, *p* = 0.063
SMA	T(11) = 2.07, *p* = 0.063	T(11) = 1.83, *p* = 0.094

	Putamen runs > SMA runs
Putamen	T(11) = − 0.27, *p* = 0.80

**Fig. 8:**
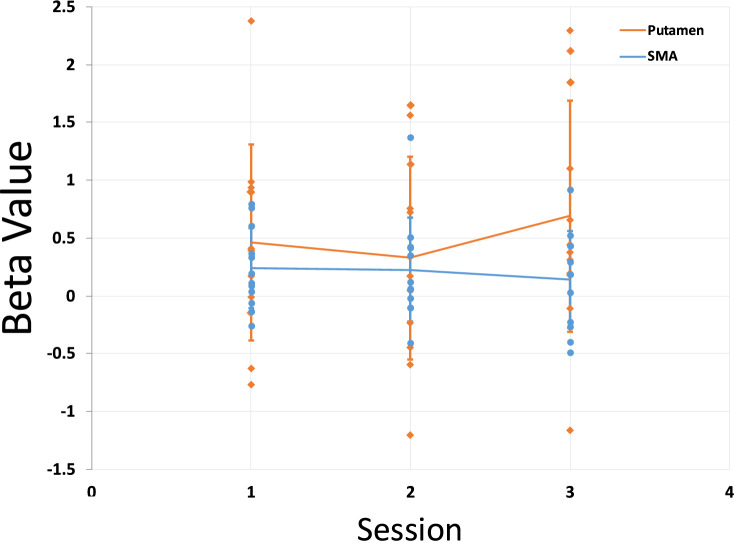
Effect of session on the NF training in the Putamen and SMA ROI conditions. The beta values represent the NF > rest condition of the ROI GLM. The scatter plot overlay shows individual betas whereas the line plot represents the average. Error bars represent standard deviation

We performed a two factorial repeated measures ANOVA to evaluate the effect of the number of sessions and ROI on NF training (Fig. 8). The analysis did not reveal any statistically significant difference of the factors session or ROI in the NF training, nor did it reveal any significant interaction [Session: *F*(2,22) = 0.43, *p* = 0.66; ROI: *F*(1,11) = 3.61, *p* = 0.08; Session*ROI: *F*(2,22) = 1.67, *p* = 0.21].

#### EMG movement control

**Fig. 9:**
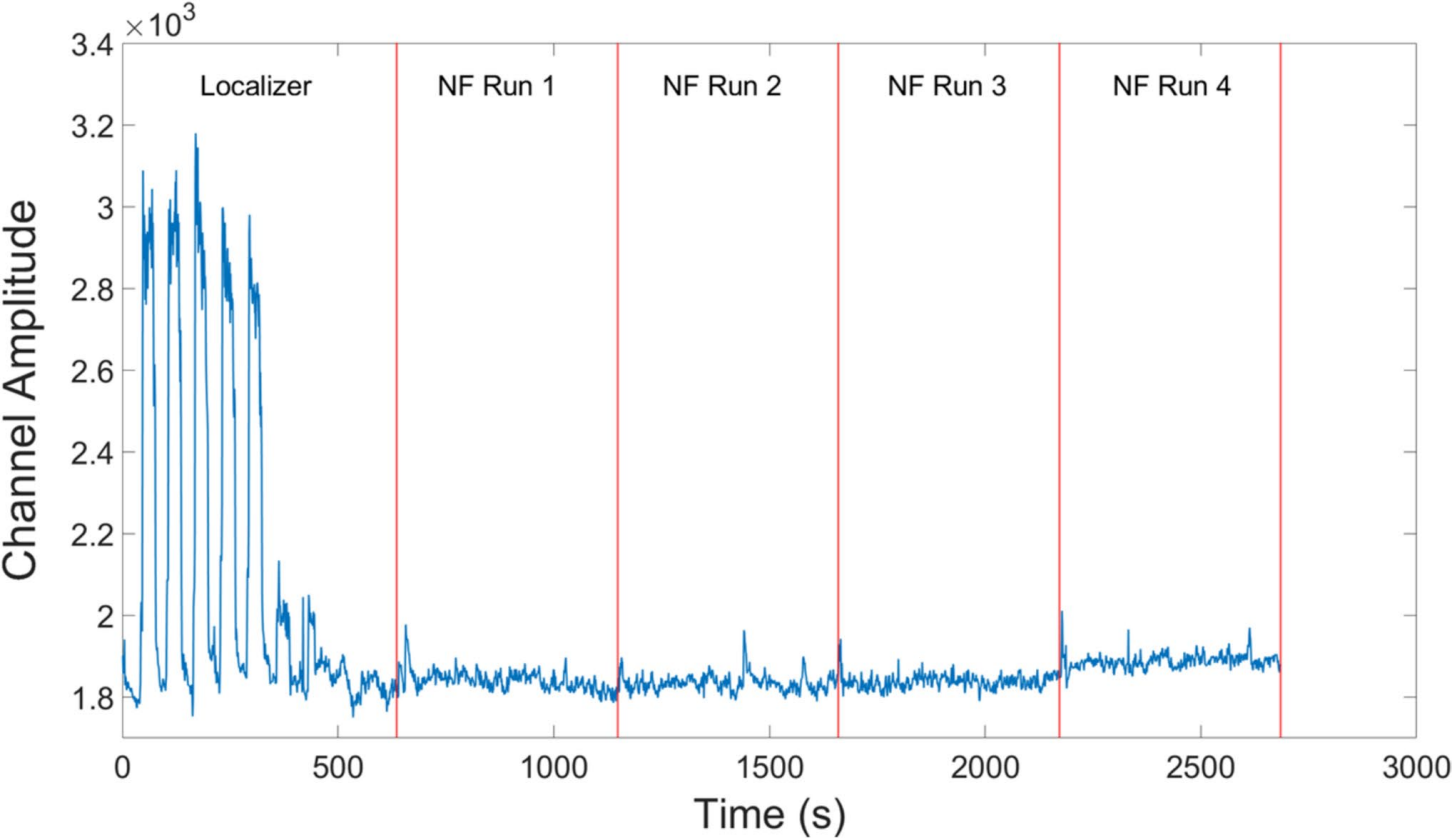
Averaged group EMG activations over all MRI sessions. The initial peaks correspond to the first five blocks of the localizer where the participants were asked to move their hands in a squeezing motion. The remaining five blocks of the localizer run as well as all the NF runs only used motor imagery as an activation strategy

**Fig. 10:**
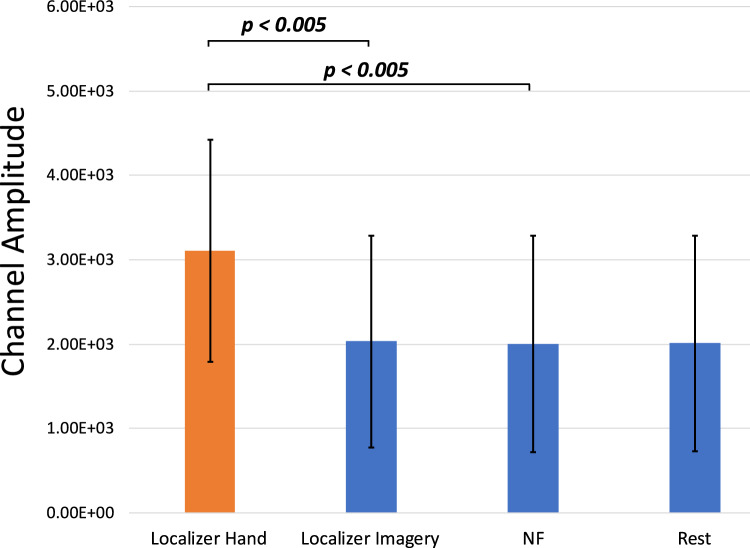
Average EMG activations over blocks within the localizer and NF runs for the patient cohort. Error bars represent standard deviation

EMG activations were extracted per participant per session and averaged across participants (Fig. 9). Due to a technical error during recording of the EMG, there was incomplete data from 5 sessions. To determine if there was a significant change in the movement during the localizer and NF tasks, we separated the EMG data into different blocks in MATLAB and then compared their average amplitudes (Fig. 10). There was a significant difference between the localizer hand movement blocks and the localizer imagery blocks as well as the NF blocks [Loc-Hand > Loc-Img: *t*(11) = 3.55, *p* = 0.0046; Loc-Hand > NF: *t*(11) = 2.57, *p* = 0.0037]. As in the healthy control group, there was no significant difference between the NF blocks and the resting blocks (*t*(11) = -0.96, *p* = 0.36).

#### Event related averages

**Fig. 11:**
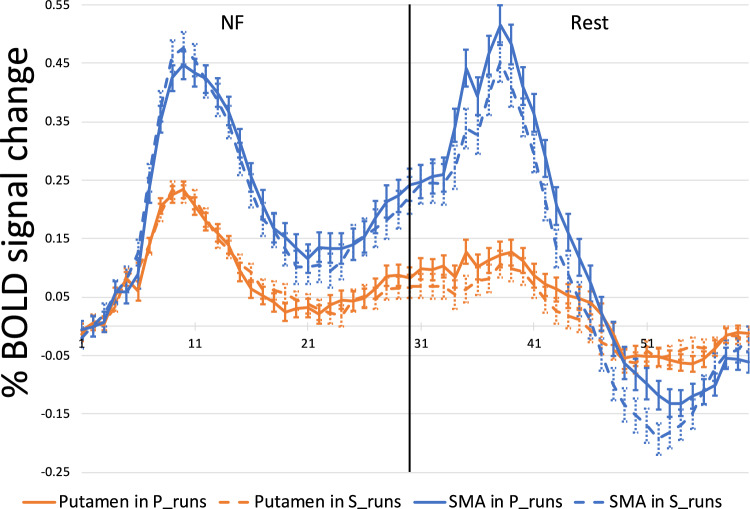
Percentage BOLD signal change during the NF blocks and the resting blocks for the patient cohort. X axis represents seconds from onset of the neurofeedback block. The blue line indicates the putamen time course during the putamen-ROI runs. The yellow line indicates the putamen time course during the SMA-ROI runs. The orange line shows the SMA time course during the putamen-ROI runs while the green line shows the SMA time course during the SMA-ROI runs. The error bars represent standard error of the mean

Similar to the healthy cohort, we extracted the ERAs for the putamen and the SMA in both conditions (Fig. 11). The NF block lasted 30 s as well as the resting block. These blocks are visually separated, and Fig. 11 shows the ERA response of the patient cohort. ERAs were extracted per participant per run while taking movement confound predictors into account and subsequently averaged per session across participants.

#### Whole brain level exploratory analysis

**Fig. 12:**
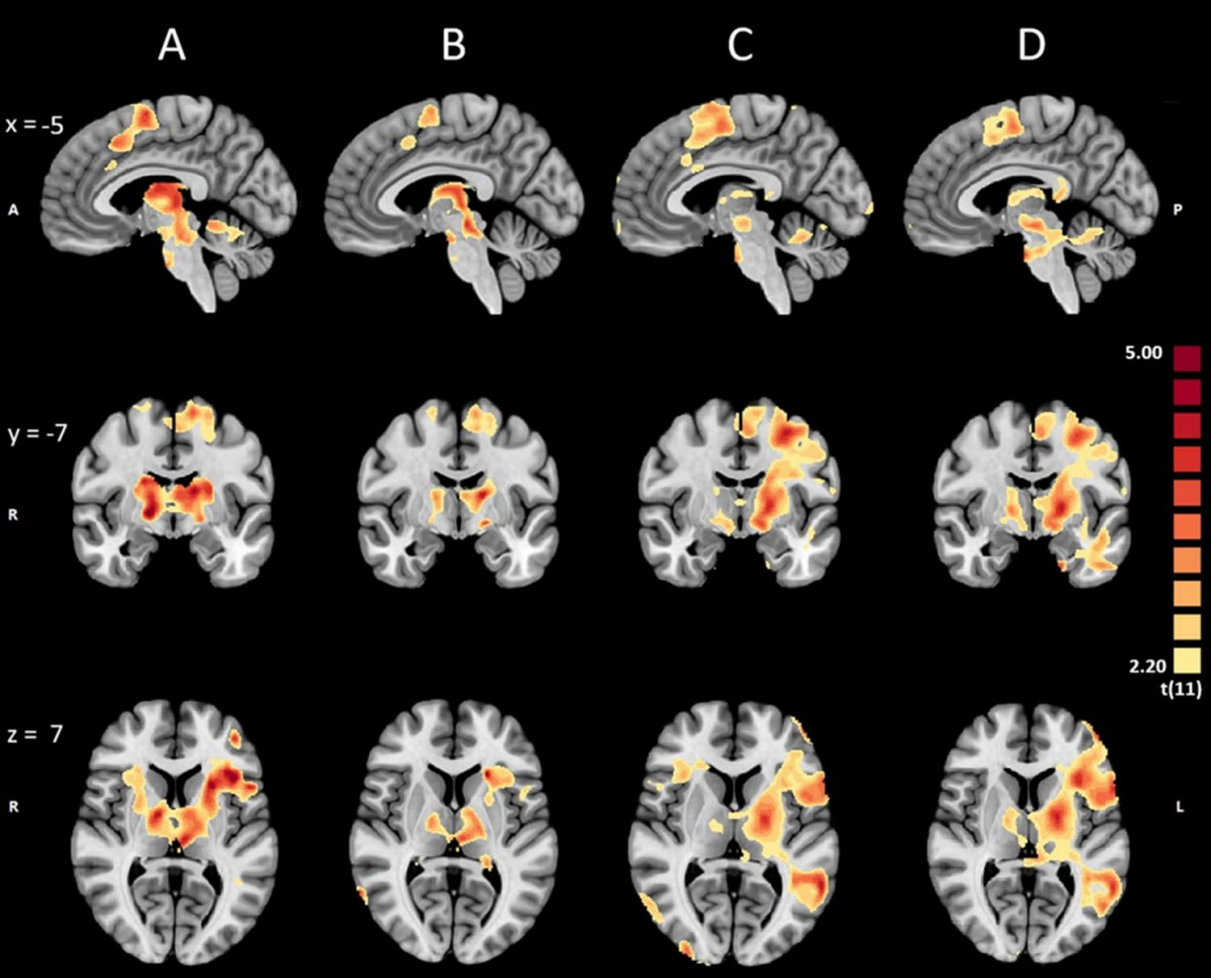
MNI brain showing: **A** Activation map in the healthy cohort for the Putamen ROI condition and NF > Rest contrast. **B** Activation map in the healthy cohort for the SMA ROI condition and NF > Rest contrast. **C** Activation map in the patient cohort for the Putamen ROI condition and NF > Rest contrast. (D) Activation map in the patient cohort for the SMA ROI condition and NF > Rest contrast. The p-value is not corrected for multiple comparisons. All contrasts have a voxel-level threshold of *p* < 0.05

An exploratory whole-brain level analysis was performed to assess the spatial coverage of the brain activations through the NF training. This analysis was performed on both cohorts and separately for each ROI condition. The T-contrast was NF blocks as compared to resting blocks in their respective condition. All contrasts shown in Fig. 12 are have a voxel-level threshold of *p* < 0.05. The p-value is not corrected for multiple comparisons. Both groups showed activation of the SMA, insula and basal ganglia (with a focus on putamen and thalamus), with a left-sided preponderance in the patient group (Fig. 12, columns C and D).

#### Cognitive strategies

After each NF run, the participants were asked to report the cognitive strategies they employed during the NF training. Their responses were recorded and broadly categorized into the following categories:

1. Exercise/Full body sport: This involved participants imagining performing physical exercises or engaging in sports that require full body exertion, such as running, swimming, playing football or working out at the gym.
2. Walking/Moving: In this strategy, the participants imagined general everyday movements such as walking, going to the store, and moving around the house.
3. Hand usage: This involved participants imagining or mentally rehearsing motor tasks or activities that primarily involve the hands, such as preparing food, gripping objects, or manipulating tools.
4. Observing movement: This strategy involved participants imagining themselves observing or watching others perform movements rather than engaging in the action themselves.
5. Singing: This strategy involved participants imagining or mentally rehearsing singing.
6. Dancing: Participants employing this strategy mentally rehearsed or simulated dancing.
7. Cognitive task: This involved participants engaging in tasks that require active cognitive processing. This could include tasks like mental arithmetic, decision-making, or meditation.

**Fig. 13:**
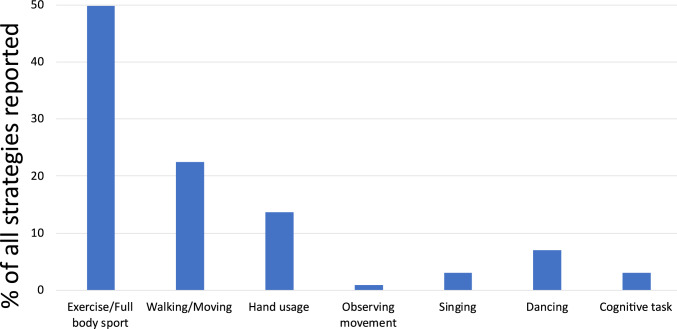
Distribution of self-reported cognitive strategies employed by patients during the NF training runs

The distribution of these strategies is presented in Fig. 13, which visualizes each category as a percentage of all strategies reported.

## Discussion

This study aimed to expand the opportunities for self-regulation training of the motor control network with fMRI-neurofeedback. It explored the viability of the putamen as a potential ROI for fMRI-based NF training in PD. Using a within-subject design we compared the recruitment of the putamen ROI with that of the SMA ROI during neurofeedback-guided motor imagery and were able to show significant activation of the targeted putamen region during NF runs in the patient cohort as well as a trend level activation in the healthy cohort.

### Healthy cohort study

Participants were able to recruit the putamen during NF training, although the observed activation reached only trend level. We suspect that this can be partially attributed to the relatively small number of participants and a single-session design, the duration of which was insufficient for participants to adequately use the NF training to develop individualized cognitive strategies (Stoeckel et al., 2014). This idea is further supported by the failed differentiation between NF and rest in the SMA ROI condition, contrary to our expectations and established literature highlighting the SMA as a successful target ROI (Park et al., 2015). In general, it seems that imagery-based activation of the motor control network was achieved, considering the relatively focused activation of SMA and motor parts of the basal ganglia documented in Fig. 11 (columns A and B). During the putamen ROI condition, we also observed significant activation in the SMA. This coactivation was expected, given that both regions are part of the motor control network (Xie et al., 2015). Finally, the putamen was equally activated in the SMA-ROI and putamen-ROI runs, further reinforcing the functional interconnectivity between the regions.

If we examine the activations of the regions in their respective ROI conditions over the time course of the NF blocks, we see that the SMA shows higher activation than the putamen. During the resting blocks, the activation patterns of the SMA and putamen diverge. The putamen appears to maintain stability and shows resilience to the transition between activation and relaxation blocks, whereas the SMA shows another increase in activation, occurring after the characteristic hemodynamic delay. This result is in line with the less specific activation patterns of the SMA (Nachev et al., 2008), which might signal any task switch, in this case from imagery to rest. This suggests that the putamen can be a better NF target candidate than the SMA since it better differentiates the NF blocks from the rest blocks. Furthermore, when observing the activation patterns of these regions for runs in which they were not the target, we see that they continue to follow a similar trajectory, consistent with their interconnected roles within the motor network (Watkins & Jenkinson, 2016).

We minimized the potential confounding effects of involuntary or voluntary physical movements by monitoring physiological activity. We saw that during the NF runs, the EMG showed no significant difference in muscle activity between the NF blocks and the resting blocks. This indicates that the participants were successfully adhering to the NF instructions without engaging in overt physical movements that could have potentially confounded the fMRI data (Friston et al., 1996; Goebel, 2021). This suggests that the EMG data served as a robust control for physical movement. The eye-tracking results indicated that there were no significant differences in the number of significant gaze movements between the NF and resting blocks. This finding suggests that participants monitored the thermometer display throughout the NF sessions, without excessive eye movements.

### Patient cohort study

We hypothesized that increasing the number of sessions would enhance the overall learning effect, providing the patient cohort with additional trials to engage in NF training and improve their ability to regulate the target regions. Additionally, given the feasibility nature of this work, the inclusion of the SMA as a positive control was intentionally retained to ensure that the putamen NF results could be contextualized against a well-studied region in motor-based NF paradigms.

Patients with PD were able to recruit the putamen during the NF blocks, highlighting its potential as a target for NF interventions in PD. We also saw that the SMA was activated at a trend level during the SMA ROI condition. Furthermore, in the case that the region was not the target ROI in the condition, both the putamen and SMA showed trend level activations. This is in line with our finding from the healthy cohort as well as the region’s functional roles in the motor (imagery) network (Haber, 2016). Similarly, we also saw that the putamen was not significantly differently activated in either ROI condition. In the patient cohort while inspecting the percentage BOLD signal change over the NF blocks for SMA and putamen targets, we see a similar activation pattern as in the healthy participant group. The difference in activation levels between the ROIs suggest that the SMA can be comparatively easier to modulate through motor imagery NF, while the putamen may require more tailored training protocols or extended sessions to achieve similar results. However, the putamen may also be more specific to actual imagery training given its lower activation during the resting period compared to the NF period.

This finding supports the growing interest in subcortical structures as targets for NF in neurological disorders. Subramanian et al. (2011) were among the first to demonstrate the feasibility of NF in PD patients, showing recruitment of the SMA with associated motor improvements. However, direct targeting of the basal ganglia, including the putamen, has been less explored in NF literature. Our study contributes to filling this gap by demonstrating that PD patients can indeed modulate putamen activity through NF, suggesting its potential as a non-invasive target for neuromodulation in PD. This is especially relevant given the role of the putamen in motor control and its relevance to the pathophysiology of PD.

This difference in activation amplitudes was expected due to the inherent difficulty of accessing a subcortical structure as a NF target region (de Hollander et al., 2017). Taking both cohorts’ temporal activation patterns together, our findings suggest a differentiation between initial effort and sustained effort. Participants generally appear capable of successfully regulating target activity at the start of the NF blocks, maintaining regulation for approximately the initial fifteen seconds. However, sustaining this effort beyond the initial phase seems to be particularly challenging. This might stem from the participants possibly trying varying strategies during the NF blocks, general habituation effects or natural difficulty in maintaining continuous regulation over longer durations.

Patients did not show substantial improvements in their ability to regulate brain activity over time. This finding is consistent with some previous studies that have reported a lack of significant session-to-session improvement in NF performance, particularly in clinical populations (Sitaram et al., 2017). Because of the limited training time and the imperative to minimize patient frustration we instructed patients to engage in motor imagery as a strategy for upregulation. These instructions may have obviated the need for initial instrumental learning and contributed to high activation levels from the outset. It has been suggested that neuroplastic changes induced by NF and further performance improvement may require more extended or intensive training protocols to manifest significantly (Stoeckel et al., 2014).

In the patient cohort, we found that the EMG results were consistent with those of the healthy cohort, showing no significant differences in muscle activity during the NF and resting blocks. With more data collected from the additional sessions in the patient cohort we can further state the robustness of the EMG recordings. This strongly suggests that the patient cohort successfully adhered to the NF instructions without engaging in overt physical movements, and confirms earlier results (Mehler et al., 2019; Subramanian et al., 2011) indicating that NF success in PD is not coupled to voluntary or involuntary movements.

### Exploratory analysis

The whole-brain analysis revealed a consistent pattern of brain activation across both cohorts during the NF training. Key regions include the supplementary motor area (SMA), insula, the basal ganglia, (particularly the putamen) and the thalamus. The SMA’s involvement is unsurprising, given its well-established role in motor planning and execution (Kornhuber & Deecke, 1965; Pfurtscheller & Berghold, 1989). The insula is associated with interoception and autonomic control, and its involvement might be crucial in achieving the desired NF performance via effective brain activations (Uddin et al., 2017). The activation of the basal ganglia structures, and particularly the putamen, even when it was not the target, aligns with their role in habit formation and reward-based learning (Ghandili & Munakomi, 2023; Watkins & Jenkinson, 2016). The observation of more lateralized activations in the patients might be a component of PD or related to side of symptom onset, however, due to the scope of this study a detailed documentation of symptom laterality was not conducted and therefore this remains conjecture. If such symptomology plays a role, it will benefit future research to possibly train the left and right putamen, and/or the SMA, independently. This approach could be particularly beneficial for the hemisphere exhibiting reduced activity, potentially yielding greater improvements in both behavioral and clinical outcomes. It is also plausible that individual differences in brain organization or cognitive strategies could contribute to such lateralized patterns in a relatively small group, such as the one in our study.

### Cognitive strategies

We saw from the self-reported cognitive strategies during NF, that the participants employed a diverse range of approaches to achieve NF success, from physical simulations like exercise, sports and dancing to cognitive approaches such as mental arithmetic and simulating conversations. This variation highlights the individual differences in cognitive processing of memory, information, training and regulation strategies. This is a key strength of the NF method which takes advantage of the plasticity of the brain and allows for a highly personalized approach for self-regulating brain activity (Loriette et al., 2021). This approach ensures that each participant can engage with NF in a way that resonates with their unique cognitive processes, maximizing the potential for success. This can be a crucial aspect, especially in patient cohorts where engagement in the applied method plays a role in determining success of the training (Mathiak et al., 2015).

### Methodological framework

The primary objective of the present study was to assess the methodological feasibility of targeting the putamen for real-time fMRI NF training in individuals with Parkinson’s disease. To this end, the experimental design was intentionally focused on the viability of the NF protocol, rather than on examining clinical efficacy or treatment outcomes. Therefore, the study deliberately minimized the inclusion of detailed clinical assessments. While these clinical parameters are undoubtedly critical in therapeutic and translational contexts, they were considered beyond the scope of this foundational investigation. This feasibility study aimed to establish methodological groundwork that could inform and enable future research to investigate the relationship between individual clinical characteristics and the capacity to regulate putamen activity through NF training.

## Limitations

Our goal was investigating the viability of the putamen as a potential NF target. This study was an essential first step in that direction and focused on the implementation of the NF method for the subcortical target. Given the methodological focus of this study, the sample size, while within an acceptable range for this purpose, represents a limitation and highlights the need for future work investigating the more mechanistic aspects of using basal ganglia as NF targets in PD patients (Tursic et al., 2020).

The study utilized a single-session design for the healthy individuals, which might not have provided sufficient time for participants to fully develop and optimize cognitive strategies for NF training. This aspect was alleviated somewhat in the patients, who had multiple sessions for NF training, as we see the activations in the SMA trend more towards our expectations from previous work. The exploratory analysis was uncorrected for multiple comparisons. With such a limited sample size it was apparent that detection of effects at the whole brain level when applying stringent corrections for multiple comparisons would not be possible. This analysis is intended solely as a preliminary indication of potential whole-brain activation patterns. It serves to inform and guide the design of future studies that are appropriately powered and structured for such comprehensive analyses.

There was no active control introduced for the NF effect in this study, and therefore, we are not able to distinguish between the effects of motor imagery and NF. No sham feedback condition was implemented as this might frustrate patients and/or exaggerate induced effects in the NF condition. Additionally, it is well established that no single control condition can comprehensively account for all the mechanisms that may influence NF outcomes (Sorger et al., 2019).

This underscores the need for larger-scale studies with calibrated session durations and frequency, along with the implementation of one or more control conditions. Such future work can assess the therapeutic potential of NF training as a non-invasive alternative for Parkinson's disease patients.

## Conclusion

This study has for the first time demonstrated the potential of the putamen as a viable neurofeedback target for patients with Parkinson’s disease. The patients successfully learned to self-regulate the putamen, suggesting its potential for individualized non-invasive neuromodulation. These findings contribute to the growing body of evidence supporting the use of neurofeedback in subcortical structures for the management of neurological disorders. Future studies with larger sample sizes, extended training protocols, and broader control conditions are essential to validate these findings and to explore the therapeutic potential of this approach in clinical settings.

## Supplementary Information

Below is the link to the electronic supplementary material.

Supplementary file1 (PDF 226 KB)

## Data Availability

The data and all related resources that support the findings of this study are available upon reasonable request from the authors.
