# Supplementary material for: Basal ganglia as an fMRI motor neurofeedback target in Parkinson’s disease
Source: Appl Psychophysiol Biofeedback. 2025 Nov 19;50(4):635–53. doi: 10.1007/s10484-025-09747-5 (PMC12685986; doi:10.1007/s10484-025-09747-5)
Supplement: Supplementary file 1 — Supplementary file1 (PDF 226 KB) [file 10484_2025_9747_MOESM1_ESM.pdf]

Supplementary Information for Basal ganglia as an fMRI motor neurofeedback target in Parkinson's disease

Baqapuri, Halim I[a,*], Terneusen, Anneke[a], Luehrs, Michael[b,c], Peters, Judith[b], Kuijf, Mark[a,d], Goebel, Rainer[b,c], Linden, David[a,d]

[a] Mental Health and Neuroscience Research Institute, Faculty of Health, Medicine and Life Sciences, Maastricht University, Maastricht, Universiteitssingel 40, 6229 ER, Maastricht, the Netherlands
[b] Department of Cognitive Neuroscience, Faculty of Psychology and Neuroscience, Maastricht University, Maastricht, Universiteitssingel 40, 6229 ER, Maastricht, the Netherlands
[c] Brain Innovation, Maastricht, The Netherlands, Oxfordlaan 55, 6229 EV, Maastricht, the Netherlands
[d] Department of Neurology, Maastricht University Medical Center, Maastricht, the Netherlands

Neuripides study group: Andres M Lozano[e], Josef Mana[f], Bechir Jarraya[g], Ricardo Loução[h], Martin Kocher[h], Veerle Visser-Vandewalle[h], Tolga Cukur[i]

[e] Division of Neurosurgery, Department of Surgery, University of Toronto, Toronto, Ontario, Canada.
[f] Department of Neurology and Centre of Clinical Neuroscience, First Faculty of Medicine and General University Hospital in Prague, Charles University, Prague, Czech Republic
[g] Cognitive Neuroimaging Unit, CEA, INSERM, Université Paris-Saclay, NeuroSpin Center, 91191, Gif-sur-Yvette, France
[h] Centre for Neurosurgery, Department of Stereotactic and Functional Neurosurgery, Faculty of Medicine, University and University Hospital of Cologne, Cologne, Germany
[i] Department of Electrical-Electronics Engineering, National Magnetic Resonance Research Center (UMRAM), Bilkent University, Ankara, Türkiye

* Corresponding author: h.baqapuri@maastrichtuniversity.nl

The pilot analysis has not been included in the manuscript due to its small sample size. Nevertheless, to support our pilot findings, we have conducted the following analysis in the healthy cohort. To investigate the MRI data, we looked at the localizer runs for all twelve healthy participants. We used BrainVoyager (BV, version 22.2, Brain Innovation, Maastricht, the Netherlands). Intensity inhomogeneity correction for anatomical images was applied before transforming them into MNI space. Standard pre-processing steps including scan-slice time correction with cubic spline interpolation and intra-session motion correction relative to the first volume of the first functional run with trilinear detection and sinc interpolation were applied. Realigned functional images were subsequently registered to the corresponding anatomical image and spatially smoothed with a 7mm Gaussian full width at half maximum kernel. We looked at the T-contrast 'Motor Imagery' as compared to 'Rest' during the localizer runs. False discovery rate correction was used at $p < 0.05$ as multiple comparisons correction. We observed significant activations in the left putamen and thalamus. No significant clusters were detected in the STN, although a small number of voxels showed activation within the GPi.

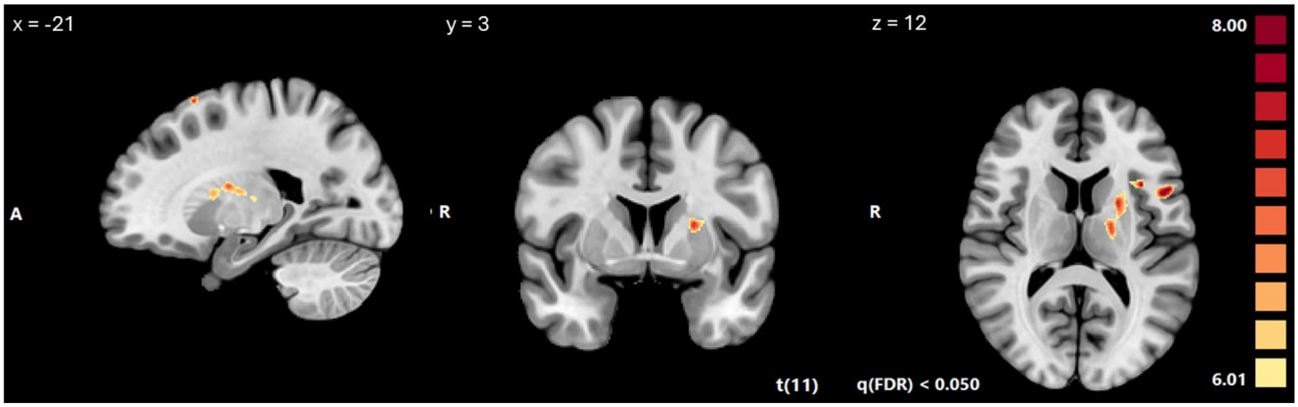

Figure 1: Activation map in the healthy cohort during the localizer runs for the contrast Motor Imagery > Rest (MNI Brain).
